# High Metabolomic Microdiversity within Co-Occurring Isolates of the Extremely Halophilic Bacterium *Salinibacter ruber*

**DOI:** 10.1371/journal.pone.0064701

**Published:** 2013-05-31

**Authors:** Josefa Antón, Marianna Lucio, Arantxa Peña, Ana Cifuentes, Jocelyn Brito-Echeverría, Franco Moritz, Dimitrios Tziotis, Cristina López, Mercedes Urdiain, Philippe Schmitt-Kopplin, Ramon Rosselló-Móra

**Affiliations:** 1 Department of Physiology, Genetics and Microbiology, University of Alicante, Alicante, Spain; 2 Helmholtz Zentrum Munich, German Research Center for Environmental Health, Analytical BioGeoChemistry, Neuherberg, Germany; 3 Marine Microbiology Group, Departament of Ecology and Marine Resources, Institut Mediterrani d’Estudis Avançats IMEDEA (CSIC-UIB), Esporles, Illes Balears, Spain; 4 Technische Universität München, Chair of Analytical Food Chemistry, Freising-Weihenstephan, Germany; Université Claude Bernard - Lyon 1, France

## Abstract

*Salinibacter ruber* is an extremely halophilic member of the *Bacteroidetes* that thrives in crystallizer ponds worldwide. Here, we have analyzed two sets of 22 and 35 co-occurring *S. ruber* strains, newly isolated respectively, from 100 microliters water samples from crystalizer ponds in Santa Pola and Mallorca, located in coastal and inland Mediterranean Spain and 350 km apart from each other. A set of old strains isolated from the same setting were included in the analysis. Genomic and taxonomy relatedness of the strains were analyzed by means of PFGE and MALDI-TOF, respectively, while their metabolomic potential was explored with high resolution ion cyclotron resonance Fourier transform mass spectrometry (ICR-FT/MS). Overall our results show a phylogenetically very homogeneous species expressing a very diverse metabolomic pool. The combination of MALDI-TOF and PFGE provides, for the newly isolated strains, the same scenario presented by the previous studies of intra-specific diversity of *S. ruber* using a more restricted number of strains: the species seems to be very homogeneous at the ribosomal level while the genomic diversity encountered was rather high since no identical genome patterns could be retrieved from each of the samples. The high analytical mass resolution of ICR-FT/MS enabled the description of thousands of putative metabolites from which to date only few can be annotated in databases. Some metabolomic differences, mainly related to lipid metabolism and antibiotic-related compounds, provided enough specificity to delineate different clusters within the co-occurring strains. In addition, metabolomic differences were found between old and new strains isolated from the same ponds that could be related to extended exposure to laboratory conditions.

## Introduction

*Salinibacter ruber* is an extremely halophilic bacterium belonging to the phylum *Bacteroidetes* that thrives in hypersaline environments. These systems are dominated by *Archaea*, such as the square-shaped *Haloquadratum walsbyi* and the recently discovered Nanohaloarchaea [1], [2], and harbour the largest number of viruses reported for aquatic systems. Since its discovery in 1999, *S. ruber* has been repeatedly isolated and/or detected in solar salterns and salt lakes worldwide in places as distant as Australia, California, the Peruvian Andes, Turkey, Tunisia and Spain [3], [4], [5], [6], [7], [8]. Many other *Bacteroidetes* that, according to 16 S rRNA gene based analysis, cluster with *Salinibacter* and apart from the rest of representatives of the group have also been detected in such environments [5], underpinning the relevance of this phylum as a main component of the autochthonous extremely halophilic microbiota in salt-saturated systems. However, so far only a few extremely halophilic *Bacteroidetes* have been brought into pure culture: *Salisaeta longa* [9], *S. ruber*, and two new species of *Salinibacter* (*S. luteus* and *S. iranicus*) isolated from an Iranian salt lake [10]. In this regard, one of the most striking results of a decade of studies of *Salinibacter* in the Mediterranean salterns from which it was originally isolated has been that all new isolates belonged to one single species.

Taxonomic studies indicated that all *S. ruber* isolates were very similar showing high phylogenetic and phenotypic relatedness [11] although they displayed a high genomic microdiversity, according to the high variability of their genomic restriction patterns resolved by pulsed field gel electrophoresis (PFGE). Indeed, in every new round of *S. ruber* isolations from water samples, new PFGE patterns were observed and in no single instance were the patterns of the original strains (used for the species description) retrieved again. When strains from different geographical origins were compared, these genomic patterns did not provide any sound indication of biogeographical segregation within the species, nor did other genetic comparisons based on RAPD and MLST [11], [12]. However, a metabolomic analysis by means of ion cyclotron resonance Fourier transform mass spectrometry (ICR-FT/MS) showed that strains of *S. ruber* isolated from different sites in the world could be grouped into three geographical areas (Mediterranean, Atlantic and Peruvian Andes) according to their characteristic metabolites [12]. More specifically, components generally associated with cell membranes, such as fatty acids and terpenoids, were responsible for the geographic discrimination.

In a subsequent study, an in-depth comparison of the two closest strains (belonging to the above mentioned Mediterranean group) was undertaken to get a deeper understanding of *S. ruber* microdiversity [13]. Strains M8 and M31, that were isolated from the same environmental sample in 1999 [14], had identical 16 S rRNA genes and ITSs and shared around 90% of their genomes although some hypervariable regions specific of each strain could be detected, that were enriched in genes coding for sulfotransferases and glycosyltransferases. Accordingly, metabolomic analyses indicated a consistent difference in sulfonated and glycosylated metabolites within the two strains, mainly in the extracellular fraction. Thus, two very close members of the same species grown under the same conditions were expressing different metabolomes.

In order to explore whether this metabolomic diversity was a general trend within the species, and get an insight of the biosynthetic potential of *S. ruber*, we have characterized the metabolomes of two sets of co-occurring isolates from two salterns belonging to the same geographical area. Metabolomics of these strains have been evaluated as a mean of unveiling the structure of the species and compared with other two well established taxonomic tools (MALDI-TOF and PFGE).

Overall our results show a phylogenetically very homogeneous species expressing a very diverse metabolomic pool. The high analytical mass resolution of ICR-FT/MS enabled the description of thousands of putative metabolites from which to date only few can be annotated in databases. Some metabolomic differences, mainly related to lipid metabolism and antibiotic-related compounds, provided enough specificity to delineate different clusters within the co-occurring strains.

## Materials and Methods

### Strain Isolation

Brine samples were collected from the salterns of “Salinas de Levante” in Campos (Mallorca) in July 2006 and “Bras del Port” in Santa Pola (Alicante) in July 2007 (with the permission of the owners). For the isolation of the autochthonous *S. ruber* strains 100 µl of the brines were directly plated onto SW 25% media (containing per litre: 195 g NaCl, 34.6 g MgCl_2_·6H_2_O, 49.5 g MgSO_4_·7 H_2_O, 0.72 g CaCl_2,_ 5 g KCl, 0.17 g NaHCO_3_, 0.65 g NaBr) emended with 0.1% yeast extract (pH 7.2). Plates were incubated at 37°C for 2 months until colonies were visible. Single isolated colonies were picked from the plate, inoculated in 1 ml of liquid SW 25% with 0.2% yeast extract, and incubated at 37°C with shaking for one week. Colonies of *S. ruber* were identified by PCR with specific primers as previously reported [15]. For this purpose, DNA extracts were obtained from 100 µl aliquots of grown cultures by centrifuging, resupending in sterile mQ water and boiling for 5 minutes. *S. ruber* isolates were further grown onto SW 25% plates emended with 0.2% yeast extract. The cultures were purified by two additional single passes of a single colony to obtain a pure culture.

### Reference Strains

Strains M31^T^ and M8 isolated from “Salinas de Levante” in Campos (Mallorca), the strains P13 and P18 isolated from Santa Pola salterns in Alicante in 1999 and the strain IL3 isolated from Ibiza salterns in 2003 [11], [15], were used as controls.

### PFGE Studies

In order to obtain enough biomass for the pulsed field gel electrophoresis, the new isolates and reference strains were grown in 20 ml SW 25% emended with 0.2% yeast extract at 37°C until the OD_600_ ranged between 0.5 and 0.6. Cells were harvested by centrifugation in microfuge tubes at 16,000 g, and the supernatant was discarded. Pellets were washed once with 800 µl of Pett IV (10 mM Tris.HCl pH 8.0; 3 M NaCl), and resupended in 500 µl of buffer. The cell suspensions were warmed at 37°C, mixed 1∶1 with 1.6% low melting agarose, and solidified in 0.1 ml molds for 15–20 minutes at 4°C. Agarose plugs were incubated overnight at 50°C in ESP buffer (EDTA 0.5 M pH 9–9.5; 1% N-laurylsarcosine; 0.5 mg/ml proteinase K). Proteinase K was deactivated by incubating the plugs in 1.5 M Pefabloc (Roche). Pefabloc was removed by washing six times for 30 minutes in TE (10 mM Tris-HCl pH 8.0; 1 mM EDTA pH 8.0). Total DNA was further digested with 30 U of *Xba*I (New England Biolabs) in a final volume of 200 µl following the recommendations of the manufacturer.

PFGE was performed in a CHEF-DRII apparatus (BioRad), using TBE 0.5X buffer, and 1% LE agarose (FMC) gels. Electrophoreses were run at 14°C using a constant voltage of 6 V/cm and a pulse ramp of 8–12 seconds for 30 h. Low Range PFGE Marker (New England Biolabs) was used as size standard. Patterns were compared using the software FPQuest (BioRad).

### MALDI-TOF MS Analyses

Matrix assisted laser desorption/ionization time-of-flight mass spectrometry (MALDI-TOF MS) analyses and data treatment were performed as previously reported [16]. The experiments were performed by the company Anagnostec (Germany) using the SARAMIS software and databases. Biomass was grown on solid medium until colonies of, at least, approximately one millimetre in diameter were visible. For MS analyses, a small amount of biomass (10^5^ to 10^6^ cells) was transferred, using a sterile pipette tip, to a FlexiMass stainless steel target. The cells were extracted on the target with 1 µL of matrix solution, consisting of a saturated solution of α-cyano-4-hydroxy-cinnamic acid in a mixture of acetonitrile:ethanol:water (1∶1∶1) acidified with 3% v/v trifluoroacetic acid. The cell suspension in matrix solution was allowed to evaporate at room temperature and crystal formation was observed. For each strain, mass spectra were prepared, in duplicate, and analyzed using an AXIMA Confidence instrument (Shimadzu/Kratos, Manchester, UK), in linear positive ion extraction mode. Strain mass spectra were accumulated from 500 shots, derived from five nitrogen laser pulse cycles, scanning the entire sample spot. Ions were accelerated with pulsed excitation, with a voltage of 20 kV. Raw mass spectra were processed automatically for baseline correction and peak recognition. The strain mass spectra profiles have been stored in the SARAMIS database reference spectra for identification (www.anagnostec.de, [17]). Samples were run twice with a time lapse of one week, in order to evaluate profile differences that may occur upon aging of the cells on the media. Duplicate samples are indicated by a _02 after the isolate number. The dendrogram of the mass spectra was obtained by single linkage agglomerate similarity (similarity matrix of identical peaks) calculations. From the identical peaks between the strain mass spectra, the similarities (%) were calculated and were used to produce the cluster branches.

### Metabolomic Analyses

The strains were grown in 3 ml of liquid SW 25% emended with 0.2% yeast extract at 37°C with shaking one week as reported for previous metabolomics studies of the species [12]. Two batches were prepared independently: the 22 strains of Mallorca salterns and the 35 Santa Pola strains, both with the same set of reference strains. Grown biomass from the two sets of culture batches was treated as previously published (12) to obtain three different extracts, extracellular (“SN1”), cell soluble or intracellular (“SN2”) and cell insoluble (“Pellet”) fractions. For metabolite extractions, 2 mL cell suspensions were centrifuged (16000 g, 2 min at 4°C). Pelleted biomass was suspended in 1 ml of Milli-Q water and sonicated to obtain a clear lysate extract. The lysate was acidified by the addition of 50 µL of 98–100% formic acid. After the acidification, the clear lysate formed insoluble aggregates that could be separated from the soluble fraction by centrifugation (16.000 g, 2 min at 4°C). The clear supernatant (SN2) was stored for further fractionation, and the insoluble pellet (Pellet) was resuspended in 500 µL of methanol and stored at −20°C until use. Both acidified extracellular and cellular soluble fractions were solid phase extracted using Bond Elut C18 columns (Varian Inc., Lake Forest, CA, USA). The retained fraction was recovered by the use of methanol [12].

### ICR-FT/MS

Broad band mass spectra were acquired on a Bruker (Bremen, Germany) APEX Qe ICR-FT/MS with a 12 T superconducting magnet and an Apollo II electrospray (ESI) source in negative and positive mode. The positive mode was selected as it showed the highest number of signals with the most annotated signals in the databases and also differentiated best in the multivariate statistics. The samples were infused in methanol with the microelectrospray source at a flow rate of 120 µL h^−1^ with a nebulizer gas pressure of 20 p.s.i and a drying gas pressure of 15 p.s.i. (200°C). Spectra were externally calibrated on clusters of arginine (10 mg L^−1^ in methanol), and calibration errors in the relevant mass range were always below 100 ppb, which is the prerequisite for an adequate elementary composition determination up to higher masses. Relative standard deviation in the intensity values of the peaks was routinely lower than 5% under our analysis conditions. The spectra were acquired with a time domain of 1 megaword (where 1 data word corresponds to 32 bits) and a mass range of 150–2000 m/z. The spectra were zero filled to a processing size of 2 megawords. A sine apodization was performed before Fourier transformation of the time domain transient. The ion accumulation time in the ion source was set to 0.2 s and 1024 scans were accumulated for one spectrum.

ICR-FT/MS spectra were exported to peak lists at a signal-to-noise ratio (S/N = 1) and they were aligned with an in-house software [18] prior to further analysis. The possible elemental formulas were calculated for each peak in batch mode by a software tool written in-house (FORMULAE®). The generated formulas were validated by setting sensible chemical constraints (nitrogen rule, atomic oxygen to carbon ratio O/C ≤(2+C2), carbon C ≤100, oxygen 0≤80, nitrogen N ≤5 and sulphur S ≤1) [19].

In order to evaluate putative patterns several visualization tools and multivariate techniques were used. With those it was possible to reduce the dimensionality of the dataset and extrapolate informative masses characteristic for the different strains. Several models were built, starting with unsupervised models as principal component analysis (PCA) and hierarchical cluster analysis (HCA). Furthermore, supervised models as partial least square discriminant analysis (PLS-DA) and orthogonal partial least square discriminant analysis (OPLS-DA) were used [20], [21], [22], [23]. The masses with the highest regression coefficients were selected as discriminative for the different phases [12]. Further extrapolation of significant masses was done using a non-parametric test (Wilcoxon test with p<0.05).These lists of masses were evaluated and assigned with the use of MassTRIX [24], [25] and the Japanese (www.metabolome.jp) metabolome databases. The statistical analyses were carried out with SIMCA-P 12.0 (Umetrics, Umea, Sweden), and SAS version 9.1 (SAS Institute Inc., Cary, NC, USA).

Network analysis was done according to Tziotis et al [26] with a metabolic “mass difference list “at 0.1 ppm edge formation error. All nodes are mass peaks assigned to empirical formulae with an error <0.5 ppm.

### Rarefaction Analysis and Diversity Indexes

The PAST software v1.82 b was used to compute the statistical diversity indexes (Shannon-Weiner) in the sequence dataset. Rarefaction curves were performed using the Analytic Rarefaction 1.3 available at http://www.uga.edu/~strata/software. Good’s coverage: *C*  = 1 -(*ni*/*nt*), where *ni* is the number of OTUs observed exactly once and *nt* is the total number of sequences.

## Results and Discussion

### Diversity of Isolates Based on Genomic and MALDI-TOF Fingerprints

From the plated samples of Santa Pola (SP) and Mallorca (RM), 35 and 22 *S. ruber* strains, respectively, were isolated. These newly isolated strains reflected a part of the cultivable population diversity of members of this species simultaneously occurring in each saltern at the time of sampling. In order to ascertain the degree of co-occurring microdiversity within the species at every location, a polyphasic approach was undertaken that included PFGE, MALDI-TOF MS and high-resolution metabolomics by means of ICR-FT/MS.

**Figure 1:**
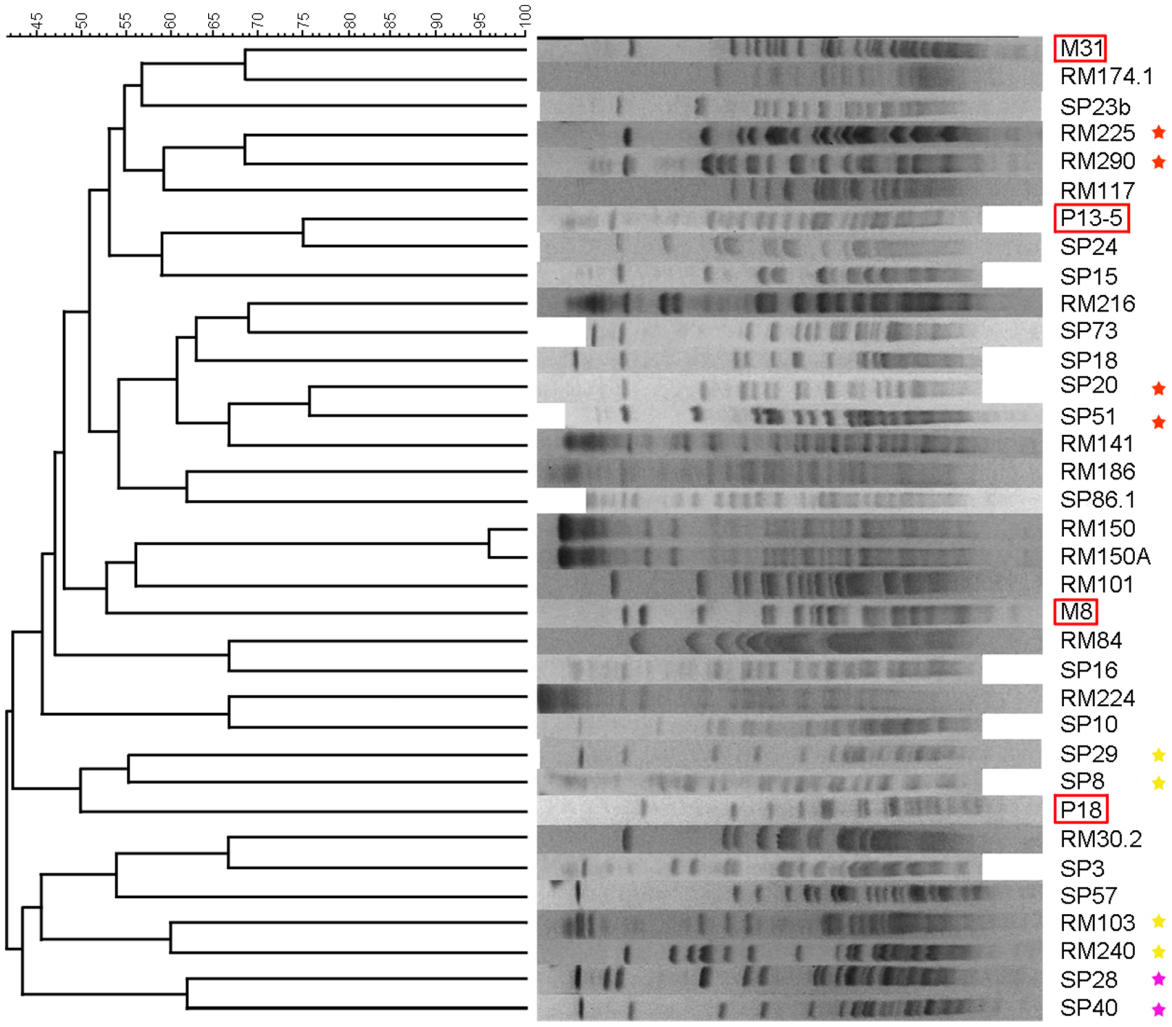
Similarity dendrogram (left) comparing the analyzed *Salinibacter ruber* strains using UPGMA analysis of their *Xba*I genomic restriction products separated by PFGE (right). Framed in red, reference strains isolated in 2000 and used here as controls. Stars mark pairs of closely related strains from the same origin for comparison with Figure 5.

Intact genomic DNAs from the strains were digested with *Xba*I and submitted to PFGE analysis yielding the profiles shown in Figure 1. The choice of the restriction enzyme was based on the high GC content (close to 70%) of *S. ruber* since this enzyme recognizes the sequence TCTAGA, which is highly infrequent in high GC genomes. Ten isolates could not be included in the analyses since their DNA was reproducibly degraded prior to restriction nuclease treatment, most likely due to endogenous nuclease activity [27] while others (around 10) could not be digested with *Xba*I, likely due to methylation of the target sites (see below). DNA from some of these strains was digested with the restriction enzyme *Ssp*I, yielding different patterns (data not shown). All the digested genomes could be resolved using the same electrophoretic conditions, which indicated their similarity, although each strain had a distinct restriction pattern. In addition, there was no clustering of the genomic patterns according to the place of isolation of strains, and RM and SP strains were mixed along the groups shown in the similarity dendrogram (Figure 1). This was also the case for the reference strains, which did not cluster with the strains isolated from the same environment several years later. Overall, the new isolates showed a level of similarity within the range previously observed for members of the species [11]. Thus, in accordance with the known genomic intraspecific diversity observed with other cultivated organisms [28], *S. ruber* patterns did not resolve any geographical common fingerprint. We cannot rule out the possibility that different strains have different levels of site-specific methylation that could result into different restriction patterns [29] although most frequently methylation results in the lack of digestibility, as observed for some strains. Furthermore, this high genomic (micro)heterogeneity has been repeatedly observed [5], [11] in the species and does not seem to be artifactual.

In parallel to the genomic approach, a phenotypic characterization using MALDI-TOF MS of whole cell extracts was carried out. This tool provides species-specific macromolecule profiles [16]. Between 80 and 100 masses ranging from m/z 3000 to 13700 were observed in the 57 strains. The similarity dendrogram based on the comparison of the fingerprints (Figure S1) showed that nearly all strains presented identical macromolecule profiles (about 75% of the strains analyzed), and all of them were grouped within a similarity of around 80%, a value that that felt within the range of the expected intraspecies diversity [16]. These results confirmed that all isolates could be identified as members of the same species [16], and did not enable us to distinguish any kind of geographical grouping among the strains. These observations are not surprising since most of the macromolecules detected are actually ribosomal proteins with a similar genealogic resolving power similar to the rRNA [30], [31]. However, although molecules other than those of the ribosome may also influence the observed phenotype [16], at this level of resolution all strains behaved identically. Finally, although it was not its main goal, the outcome of the MALDI-TOF analyses validated the specific method of isolation and fast identification of *S. ruber* strains used here.

Thus, the combination of PFGE and MALDI-TOF provides, for the newly isolated strains, the same scenario presented by the previous studies of intra-specific diversity of *S. ruber* using a more restricted number of strains: the species seems to be very homogeneous at the ribosomal level while the genomic diversity encountered was rather high since no identical genome patterns could be retrieved from each of the samples. In order to explore this intriguing microdiversity, a detailed metabolomic analysis was carried out to try to understand the nature of the differences among the different strains.

### Metabolomic Analyses: Old Versus New Strains

Our initial studies on the metabolomic-geographical patterns of *S. ruber* showed that the isolates of different geographical areas could be distinguished based on their metabolomic profiles [12]. Here, the goal was to investigate metabolomic similarities and differences among co-occurring strains as a proxy of the “meta-metabolome” of the species in the natural environment. In addition, the analysis was carried out with two sets of isolates from two different Mediterranean salterns, geographically very close, to explore the putative differences in metabolite composition that could be identified as specific of either system.

In spite of the power of spectrometry-based metabolomics in systems microbiology [32], its use in instraspecific diversity studies has been very scarce and mostly limited to targeted metabolite analysis as for e.g. lipids [33]. Krug et al. [34] for instance, compared the metabolomes of 98 *Myxococcus xanthus* strains isolated from 78 locations and including 20 cm-scale isolates from one location and enabled to identify a number of candidate compounds, most of them polyketides or nonribosomal peptides, that greatly exceeded the number of metabolites previously known as produced by this species.

In this study we describe the metabolite diversity using non-targeted mass spectrometry based metabolomics with the two sets of strains (22 RM isolates and 35 SP isolates, respectively) that were independently cultured; in both cases the same reference strains (M8, M31^T^, P13, P18 and IL3; 15) isolated 8 years earlier were included. For both experimental sets, we used the same media composition as well as the same culturing conditions (time of incubation, tube type, shaking speed, temperature, etc…). The analysis of the metabolomic profiles for each strain was carried out as in previous experiments considering extracellular, intracellular and methanol extracts of the pellets after centrifugation [12], [35].

The chemical space can be described and visualized in metabolite networks for an overall vision of the global results and be analyzed in correlation networks (Figure S2A, B) confirming thus the results of the classical non supervised statistics [Figure S3] indicating that the three cellular fractions (supernatant “SN1”, intracellular soluble “SN2”, and intracellular soluble “Pellet”) have different molecular fingerprints. The chemical diversity of 3135 putatively annotated signals is shown in Figure S2A, where nodes represent masses and connections (edges) are mass differences which represent potential biochemical reactions. Signals which were representative for the cellular classes were colored as follows: extracellular: blue, intracellular: green, pellet: red. Extracellular compounds cluster in five distinct modules while two large intracellular modules and three distinct pellet modules can be observed. One intracellular module is largely mixed with extracellular compounds, which indicates close compositional relationships. Both intracellular modules are connected via three of the extracellular modules and some pellet compounds. This topology indicates the metabolic probably pathway-based involvement of the cellular classes. The rather apical position of two pellet compounds underlines the chemical exclusiveness of cell-wall components. One large extracellular module is rather distant to the central modules, which indicates exclusive extracellular chemistry. Thus, Figure S2A indicates two central metabolic clusters where one is closely associated to ‘cell wall metabolism’ and the other is associated with trans-cell-wall metabolite fluxes.

In Figure S2B we used a correlation network approach in order to evaluate whether geographical classes as they were verified through statistical analysis would cluster correspondingly. We constructed a metabolic correlation network in which the nodes represent the 186 samples (the three fractions for the 86 strains analyzed), and the edges represent the positive Pearson correlation coefficients calculated between the samples (Figure S2B). The network of Figure S2B was coloured corresponding to Santa Pola (red), Mallorca (blue) and reference strains (green). The upper network entirely consists of Pellet samples. Both, the Santa Pola strains and for Mallorca strains exhibit a two modules in the lower network. The left module consists exclusively of extracellular samples, while the right module is composed from intracellular samples.

In this regard, SN2 and pellet were quite homogeneous for the analyzed strains, whereas SN1 (extracellular) data were more disperse, indicating a higher diversity within this fraction. SN1 includes all the metabolites present in the culture media (that have been used to different extents by the different strains) and all the metabolites secreted by the strains to the media. This highly variable set of metabolites requires the presence (either for import or export) of a highly diverse pool of transporters. Indeed, this seems to be the case within the species *S. ruber* as indicated by the study of its environmental pangenome [36]. These authors found that the metagenomic islands of *S. ruber* (i.e. fragments of the genome displaying high variation within different members of the species) were enriched in substrate transporters. Furthermore, *Haloquadratum walsbyi*, the archaeon that dominates most of the environments where *S. ruber* thrives [37], also presents a rich pool of transporters within its pangenome, which is also pointing to the presence in the salterns of a highly heterogeneous dissolved organic carbon pool, a fact that, albeit indirectly, is also related with the high metabolomic diversity of SN1.

An astonishingly diverse extracellular metabolome has been recently detected by means of nanospray desorption electrospray ionization mass spectrometry when sampling living colonies of different bacterial genera [38]. In the words by Taxter and Kolter [39], “the chemical landscape inhabited and manipulated by bacteria is vastly more complex and sophisticated than previoulsy though”. However, the high metabolomic diversity within the SN1 fraction of the newly isolated *S. ruber* (13530 ICR-FT/MS features, possible 7634 elementary compositions), although interesting from an ecological point of view, hampered any comparative analysis between the strains since practically each of them behaved in a specific manner, and no clustering of similarity trends could be detected among them.

**Figure 2:**
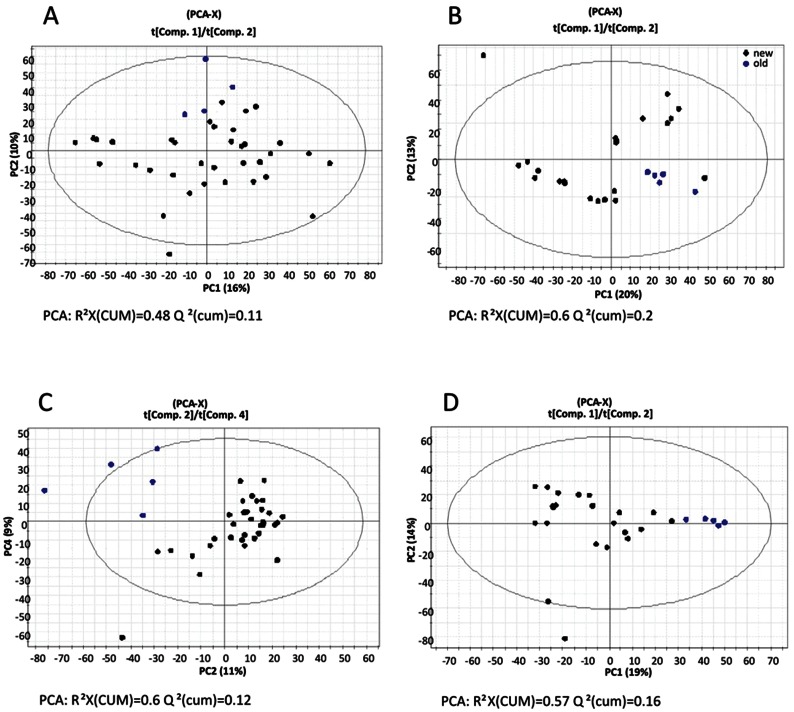
Diagrams based on unsupervised PCA analysis of all metabolites present in the cellular soluble fraction of the new isolates (black dots) and old isolates (blue dots) showing the relative homogeneity of the old isolates. (A) PCA of the cellular soluble fraction (SN2– SP) of the experimental set of Santa Pola isolates; (B) PCA of the cellular soluble fraction (SN2– RM) of the experimental set of Mallorca isolates; (C) PCA of the cellular insoluble fraction (Pellet – SP) of the experimental set of Santa Pola isolates (in this case the best distribution was observed with the components 3 and 4; and (D) PCA of the cellular insoluble fraction (SN2– RM) of the experimental set of Mallorca isolates.

In addition, unsupervised PCA of the metabolomic profiles indicated that although both experiments were carried out under the same conditions, the two sets of control strains did not behave identically (Figure S4). For this reason, both experimental sets were analyzed independently in order to avoid incongruence. However, in spite of these differences between the two datasets, the four generated PCAs (Figure 2) showed consistently that in all cases the metabolomic profiles from old strains presented an homogeneity that distinguished them from the rest of the strains under study. It was remarkable to see that the old strains isolated from Mallorca (M8 and M31^T^) were more similar to old isolated from Santa Pola (P13 and P18) than to new isolates of the same origin.

**Figure 3:**
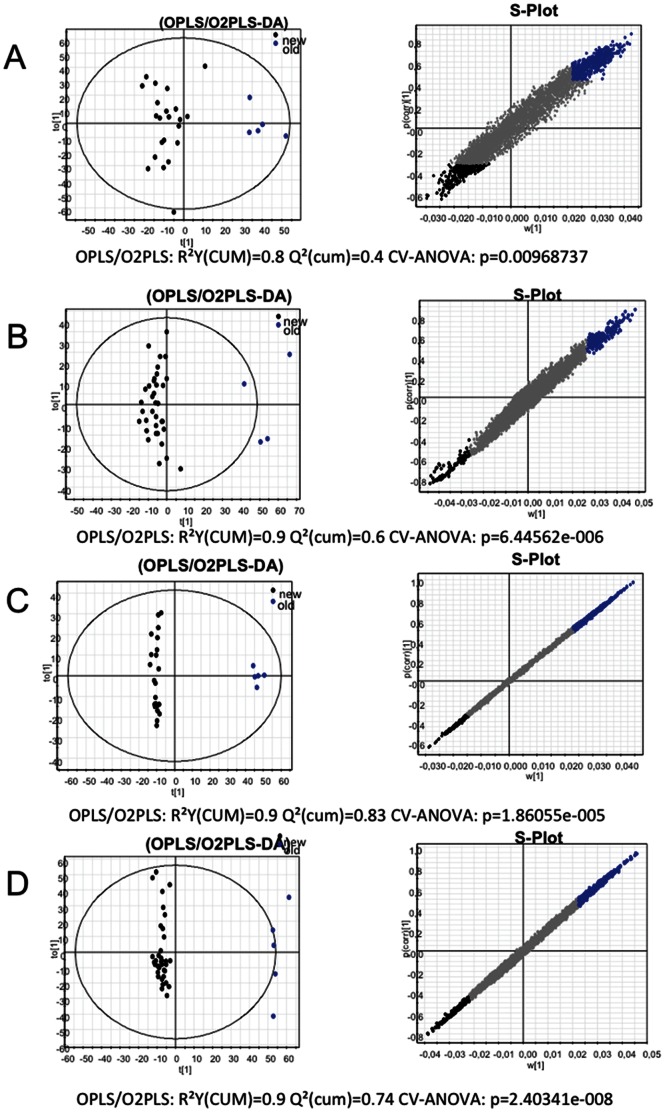
Left column: diagrams based on supervised OPLS analyses showing the discrimination of the different isolates old (blue dots) and new (black dots) of the fractions: cellular soluble fraction of Mallorca new isolates SN2-RM (A) and Santa Pola new isolates SN2- SP (B); and cellular insoluble fractions of Mallorca new isolates Pellet-RM (C) and Santa Pola new isolates Pellet- SP (D). Grey dots represent the core metabolome of all isolates that do not offer any discrimination. The selection of the compounds is done taking in account the different magnitude of correlation and covariance. The highest value for each list is associated the value 100%. This is the reference percentage and all other values are scaled accordingly to it. The value that present both value of percentages above 50% have been assumed to be a candidate that can be investigate in MassTRIX, the percentage has been set up as a cut-off of the data.

In order to retrieve the masses responsible for the discrimination between old and new strains in each experimental set, we performed a supervised OPLS analysis (Figure 3). From the analysis we could observe that the global metabolome for each fraction and experiment ranged from 3700 to 5700 masses (Table 1). Between 26% and 7% of the masses were responsible for the discrimination of old and new isolates metabolomes, whereas the rest of the metabolites made up the core metabolome of the strains in study (grey dots in Figure 3). The molecular masses were transformed to CHONS, and further annotated using the MassTRIX web server using the KEGG database with the *S. ruber* M31^T^ and M8 genomes as reference. From all discriminative masses, just between 14% and 17% could be annotated (Tables 1 and S1), whereas the rest remained unknown (but not less relevant) based on the current knowledge on the *S. ruber* metabolic pathways. This low proportion of annotable metabolites within the metabolome is mirrored by the high proportion of hypothetical proteins encountered when annotating the first microbial genomes sequenced, both issues being related to database limitations. As pointed out by Watrons et al. [40], “most molecules involved in metabolic exchange are unique to one or a few organisms” and thus there is no currently available neither knowledge nor database covering this plethora of different metabolites, even for the best known microbes. Only the empirical approach using non-targeted metabolomics with (ultra)high resolution analytics combined with adequate mathematics and bioinformatics will enable us to describe this chemical diversity and unravel, step by step, putative new structures of secondary or whole classes of metabolite conjugations never described before.

**Table 1:** Number of metabolites responsible for the discrimination between old (reference strains) and new isolates for each experimental set.

Fraction	Total metabolome^1^	Total discriminative masses^2^	Total annotable masses^2^	Metabolites with assigned pathways
SP (SN2)	5742	410 (7%)	71 (17%)	56
		274 (old)	51 (old)	
	(2705–5077)			
		136 (new)	20 (new)	39 (old)
SP (PELLET)	4628	661 (14%)	94 (14%)	
		413 (old)	65 (old)	17 (new)
	(2009–2871)			
		248(new)	29 (new)	
RM (SN2)	4889	951 (19%)	149 (16%)	96
		508 (old)	76 (old)	
	(2343–2932)			
		443 (new)	73 (new	36 (old)
RM (PELLET)	3698	986 (26%)	140 (14%)	
		674 (old)	73 (old)	60 (new)
	(1362–2327)			
		312 (new)	67 (new)	

The number of masses corresponds to those identified as CHONS in where the isotopes had been removed. SN2 refers to the cellular soluble fraction, and PELLET to the cellular insoluble fraction. Annotable masses refer to those whose molecular formula could be assigned to a given metabolite using *Salinibacter ruber* strains genomic data.

1- Total number of masses in the corresponding fraction for the analyzed strain; in brackets, the range of metabolites found in the different strains.

2- In brackets, the percentage of masses of the discriminative metabolome, and the corresponding set of strains set of strains (new) or (old) respectively.

**Figure 4:**
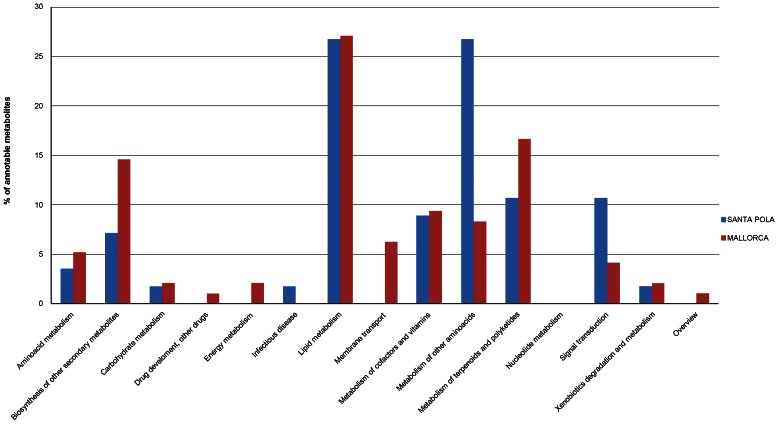
Distribution in the different metabolic classes of the annotable metabolites responsible for the differences between old and new isolates in each of the analyzed datasets.

For both datasets, old strains synthesized the highest number of discriminative metabolites; in other words, the overall number of metabolites that could be detected in the cultures was higher for old than for new strains. When comparing old versus new strains for each dataset, some metabolic classes were enriched in discriminative metabolites, although only a few metabolites were common to the different subsets of strains (Table S1), which again underlines the high metabolomic diversity within the species. In both datasets, the metabolic classes that included the highest number of metabolites assigned to pathways were lipid metabolism, metabolism of other amino acids, biosynthesis of other secondary metabolites and metabolism of terpenoids and polyketides. The percentage of discriminative metabolites within each class was different for Santa Pola and Mallorca datasets, as was whether old or new strains accumulated more metabolites in a given class (Figure 4 and Table S1) so it could not be ascertained that a given class was depleted or enriched in metabolites when comparing new and old strains.

In both datasets, metabolic pathways (Figure S5) related to membrane components, and included in the class “Lipid metabolism”, accumulated the highest number of metabolites discriminating between old and new strains, suggesting that cells undergo changes in their envelopes as a results of their “adaptation” to laboratory conditions. In addition, some pathways including metabolites of potentially applied interest, such as antibiotic or polyketide products, accumulated a relatively high numbers of annotable discriminative metabolites. This is indicating that the time of storage of strains under laboratory conditions may modify their biosynthetic capabilities, a finding that could be of relevance for the search of new active compounds of commercial interest out of existing biobanks. In addition, differences between old and new strains could be due to differences at the genomic level between old and new strains isolated from the same ponds, although this would not explain why old strains of different geographical origins cluster together, as observed here according to their metabolomics profiles.

### Exploring the Metabolomic Microdiversity

**Figure 5:**
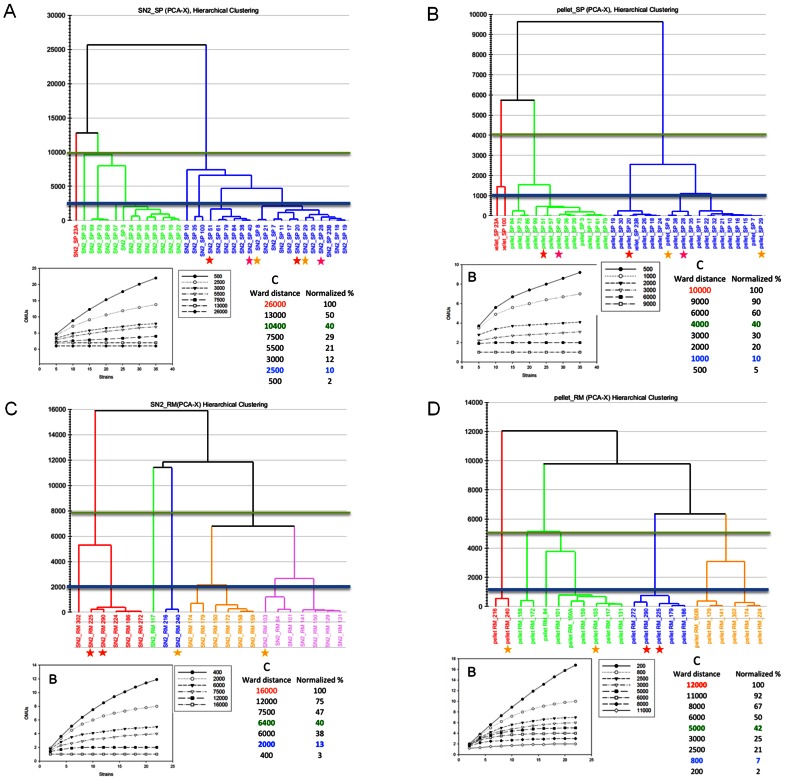
Distance dendrograms showing the similarity groups based on the pairwise comparison of the following metabolomes: cellular soluble (SN2) fraction from Santa Pola strains (A); cellular insoluble (pellet) fraction from Santa Pola strains (C); cellular soluble (SN2) fraction from Mallorca strains (C); and cellular insoluble (pellet) fraction from Mallorca strains. The blue line indicates the 10% clustering threshold (see below) for which no discriminant statistical model could be found to support the groupings observed. The green line indicates the 40% clustering threshold for which a good statistical support based on metabolite differences could be found. Diagram (B) shows the rarefaction curves based on distinct clustering threshold. Table (C) shows the correspondence of the Ward distance of the dendrogram with the percent of clustering of the strains. We take 100% clustering as a Ward distance of 26000. In green it is indicated the 40% threshold for which the grouping has statistical support, and in blue that of 10% for which no support was found. Some strains are marked with stars for comparison with Figure 1.

Since the old strains used as controls did not show identical metabolomic profiles in the two experiments, the metabolomic diversity of new strains from Mallorca and Santa Pola was studied independently. In order to investigate the putative similarities among the strains, their metabolite profiles were analyzed as described in the Material and Methods section. The results rendered a Ward distance matrix that was used to calculate a distance dendrogram for each group of strains and cellular fraction (Figure 5A to 5D). Ward’s method tries to keep minimum the square error sum when clusters are merged. The different plots gave different maximal Ward distances between all the strains (26000, 10000, 16000 and 12000 for SP-SN2, SP-pellet, RM-SN2 and RM-pellet, respectively). In order to compare them, distances between these four samples were normalized by calculating the corresponding percentages (see insets in Figure 5A to 5D). Strains were grouped by setting different distance percentage thresholds and considering the corresponding clusters of strains as different OTUs (denoted further in the manuscript as “*metabotype* based OTUs”: *m*-OTUs). For each dendrogram in Figures 5A to 5D, rarefaction curves as well as the Shannon-Weiner diversity and Good’s coverage indexes were calculated. Rarefaction curves (insets in Figure 5A to 5D) showed that at 10% clustering distance the number of *m-*OTUs already reached a plateau indicating saturation, in accordance with the Good’s coverage (Figure S6) that was close to 100% at 40% clustering distance. As expected, Shannon-Weiner indexes decreased with increasing clustering distance percentages, approaching 1 already at 40% clustering distance. These results indicated that the distance range most appropriate to analyze the metabolic diversity was between 10 and 40%. Further analysis, by using PLS-DA, of the grouping at 10% (i.e. optimal rarefaction and diversity indexes) and 40% (upper limit of resolution for diversity studies) showed that only the clustering at 40% was statistically supported to build classification models. In other words, there was a statistically solid clustering of strains at 40% Ward distance, which allowed the identification of metabolites that could be used to discriminate among the different clusters of strains. This metabolomic clustering did not show any relationship with the genomic similarities based on PFGE restriction patterns, since strains that appeared very close based on their genomic patterns (marked with stars in Figures 1 and 5), were not normally members of the same clusters.

In order to retrieve the masses of such “discriminative” metabolites, we took in consideration for each m/z value their importance in the projection (VIP). These values summarize the overall contribution of each metabolite to the classification model, and are considered significant when above 1 [41]. As shown in Table 2, from 31.2 to 43.1% of the metabolites in the different fractions were responsible for the observed clustering. Depending on the fraction considered, from 60 to 70% of the metabolomes, could thus be considered the core set of metabolites that were present with the same intensity in the same cellular fraction from all strains simultaneously isolated from the same pond. As happened when comparing old *versus* new strains (see above), only a relatively small fraction (between 6.9 to 20.5%) of the masses identified as discriminative could be annotated, whereas the rest of metabolites were of unknown nature. Around 40% of annotable discriminative metabolites were common to different fractions from strains from Santa Pola and Mallorca, which indicated that some common trends could be observed between them in terms of the “accessory” metabolomes.

**Table 2:** Number of metabolites responsible for the discrimination among the different metabolic types observed within each set of new strains of Santa Pola and Mallorca.

Fraction	Total metabolome	Discriminative metabolites (%of the total)	Annotable metabolites (% of the discriminative)	Metabolites with assigned pathways (% of the discriminative)
SP (SN2)	5742	1994 (34.7%)	294 (14.7%)	107 (5.4%)
	(2705–5077)			
SP (PELLET)	4628	1443 (31.2%)	99 (6.9%)	22 (1.5%)
	(2009–2871)			
RM (SN2)	4889	2106 (43.1%)	358 (17.0%)	124 (5.9%)
	(2343–2932)			
RM (PELLET)	3698	1551 (41.9%)	318 (20.5%)	112 (7.2%)
	(1362–2327)			

The candidate discriminative metabolites have a VIP value equal or greater than 1.

**Figure 6:**
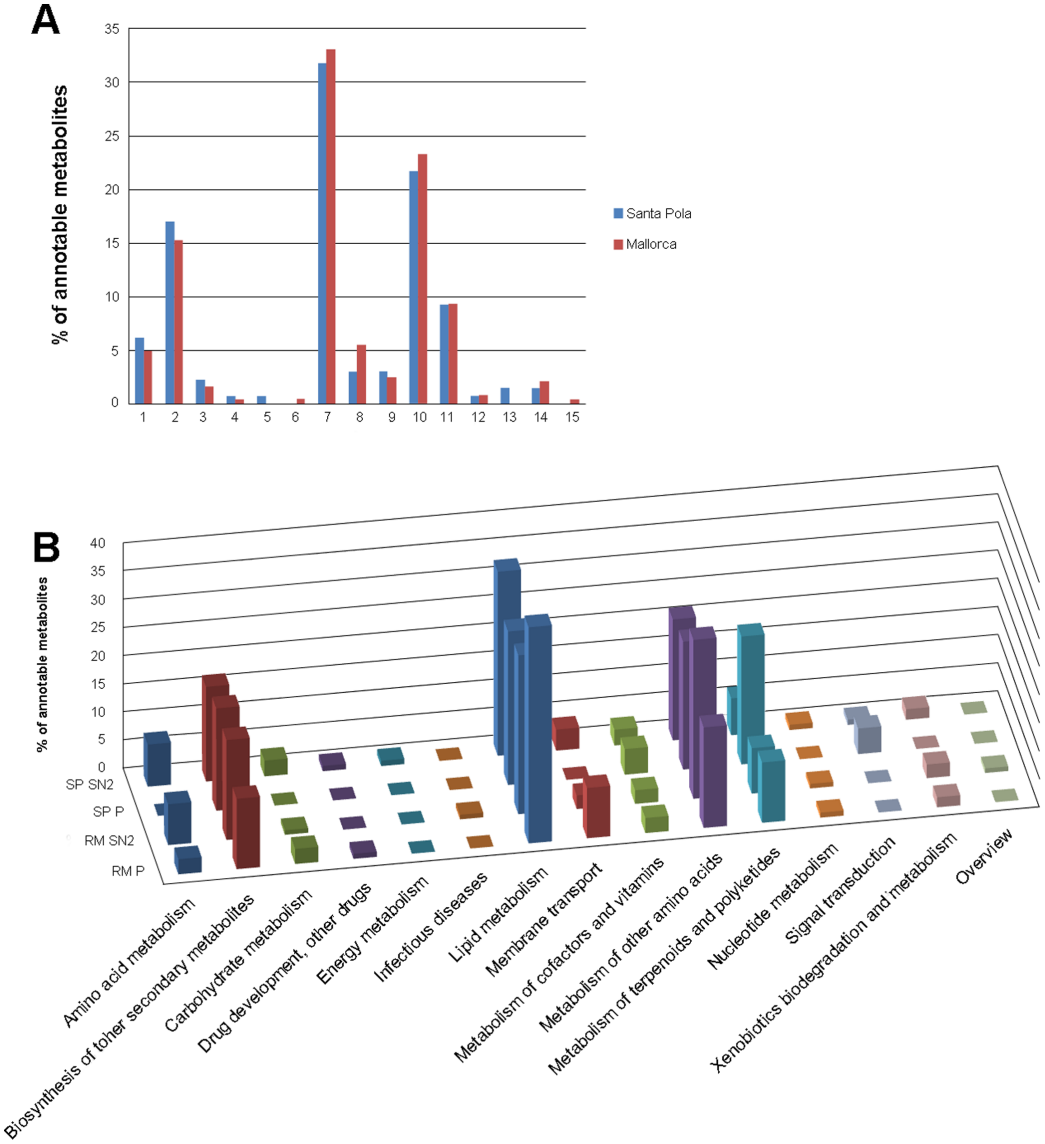
Distribution in the different metabolic classes of the annotable discriminative metabolites responsible for the clusters shown in Figure S5. Data from all co-occuring strains (A) and from the different fractions (B).

The distribution of annotable discriminative metabolites within metabolic classes was similar for both Mallorca (RM) and Santa Pola (SP) strains (Figure 6). The classes including the highest number of metabolites were, in this order, lipid metabolism, metabolism of other amino acids, biosynthesis of other secondary metabolites and metabolism of terpenoids and polyketides. These classes also accumulated the highest number of discriminative metabolites when old and new strains were compared (see above), although with a different distribution in the pathways (compare Figures S5 and S7). Within the lipid metabolism class, the pathways (Figure S7) ether lipid metabolism, fatty acid biosynthesis and sphingolipid metabolism accumulated the highest number of metabolites responsible for the differences among strain clusters. Lipid metabolism seemed to be the most versatile metabolic network in *S. ruber*, as also indicated by the wide diversity of lipids found among the discriminative metabolites in other metabolic classes (data not shown). In addition to the relevance of lipids as active components of the cell and outer membranes, lipid synthetic pathways provide precursors for the synthesis of many cellular components [42].

Within the class “Metabolism of other amino acids”, the pathway cyanoamino acid metabolism had the highest number of discriminative metabolites, followed by glutathione metabolism. Some metabolites in the cyanoamino acid pathway are also annotated as taking part in different lipid metabolism pathways and are themselves different types of lipids which, again, underscores the versatility of the lipidome. The cyanoamino acid pathway is connected to different standard and unusual amino acid pathways, including sulfur metabolism with glutathione (GSH), as well as with the nitrogen metabolism pathway. GSH is a low molecular weight thiol that is involved in bacterial redox regulation and and adaptation to stressors [43]. The GSH metabolism pathways includes different amino acids and di and tri-peptides intermediates that can change their concentrations depending on the environmental conditions [44].

The class “Biosynthesis of other secondary metabolites” includes many annotated isomers belonging to the biosynthetic pathways of antibiotics such as novobiocin, puromycin and penicillin, and cephalosporin. In addition, there is a relatively high number of metabolites in the biosynthesis pathways of biosynthesis of type II polyketides (pathways Biosynthesis of type II polyketides backbone and products, included in the class “Metabolism of terpenoids and polyketides”); metabolites derived from polyketydes are also antibiotics or are involved in defense mechanisms [45]. However, *S. ruber* M8 and M31 lack key (annotable) enzymes in the PKSII pathways which makes this finding very intriguing and worth of further studies. The production of antibiotics is a key factor in structuring the social cohesiveness in ecologically defined bacterial populations in *Vibrio* species [46]. Such populations are defined as “phylogenetic clusters of closely related but nonclonal individuals which share common ecological associations”. Thus, according to this definition, all the analyzed co-occurring *S. ruber* isolates studied here are most likely members of the same “population” although whether antibiotics and/or signaling compounds are mediating competition within the population or with potential competitors is unknown. In any case, antibiotic-related metabolites are important in structuring the differences among *S. ruber* strains and further studies are needed to understand their social behavior on a molecular basis.

#### Strain level metabolic diversity

In order to get a closer look at the metabolomic diversity among different strains, we have analyzed in detail the metabolomics profiles of strains RM84, RM101, RM 117, RM131, and RM158, all of them belonging to the same m-OTU according to their pellet composition (Figure 5). Although these five strains could not be distinguished by the statistic tools described above, their metabolite profiles are still clearly different and include compounds that are present only in a given strain. For instance, there are 88 annotable metabolites (44 in SN2 and 44 in the pellet fraction) that are only present in RM84 and absent from the rest (data not shown). The high diversity of such compounds and their unexpected nature are remarkable. Around one third of the RM84 specific metabolites were lipids but there were also antibiotic-related products, alkaloids and analogues to phytochemicals, among others. Annotation of metabolites to their isomers is limiting when using (ultra)high resolution mass spectrometry approaches, which certainly do not enable the structural identification of all the metabolites present in a complex sample (although compounds and related pathways can be proposed with high consistency). Even considering these limitations, the comparison of the chemical diversity on an elementary composition level clearly shows that very close strains (all the strains analyzed are in the same m-OTU and were isolated from the same location) show very different metabolomic potentials. Therefore, the search for new bioactive substances of pharmacological and/or biotechnological interest from microbial strains should not be restricted to the analysis of a single strain of a given species out of a biobank but rather involve on site sampling taking advantage of the highest metabolite diversity. This could be shown as well by an intraspecific metabolomic diversity analysis carried out with *M. xanthus* strains [34].

### Concluding Remarks

This work shows that very closely *S. ruber* strains co-occurring in the same environment and grown under the same lab conditions are expressing diverse metabolomes. Their comparison provides some clues to establish clusters within the species, as well as to separate new strains form the old strains isolated from the same salterns 8 years before and used for the species description. This high metabolomic microdiversity within the new isolates, as well as their diverse PFGE patterns, is very intriguing considering the, apparently, low opportunities for micro-niche differentiation offered by the waters from crystallizer ponds, where *S. ruber* lives. However, this vision of the water salterns as completely homogeneous medium can be artifactual given the new view of the micro-architecture [47] and microbial networks [48] in marine waters. In addition to these putative spatial discontinuities, *S. ruber* strains are exposed in nature to the strain-specific attack of viruses [13], [49] and to changes in environmental conditions due to seasonal dynamics and saltern operations [50]. The great metabolomic variability observed among co-occurring individuals of *S. ruber* in a small brine volume may be an indication that the environmental microbial niche diversity is far beyond our current understanding.

## Supporting Information

Figure S1 **Dendrogram based on the Maldi-Tof profiles of all strains used in this work.** Duplicates analyzed after one week of culture incubation are indicated by a _2 in the dendrogram. A group of 3 *Halococcus* sp. (Pardela 6 to 8) have been used as out-group for the analyses, as well as some additional unidentified isolates from S’Avall salterns (Mallorca) had been used as internal controls. S’Avall saltern isolates are indicated with the prefix SA
(TIF)

Figure S2 **A**: Mass Difference Network created from all annotated data Node Colors: Blue: Extracellular, Green: Intracellular, Red:Pellet. Colors were given only if the frequency of an m/z peak was 10 fold higher than the average mass frequency within the other classes. **B**: Correlation network created at threshold 0.90. Each nodes represent a sample; the closest the highest the correlation Regions are labeled as follows: extracellular blue, intracellular green, and pellet red. This approach individualized class specific subnetworks confirming thus the PCA grouping of the sample presented in Figure **2**.
(TIF)

Figure S3 **All the data together, in blue the different control samples and in red the new strains (A) As an example, the analyses for the Santa Pola isolates are shown: SN1 (B) SN2 (C) and pellet (C).** Score scatter plots of all the isolates (A) from these we spit the data in 3 datasets to visualize that the dispersion of the control samples in SN1 (B) is greater than in the other datasets(C, D). 2012.
(TIF)

Figure S4 **Unsupervised PCA analysis of each fraction (SN2 (A) and Pellet (B)) taking into account all samples obtained.** Red triangles indicate the new strains of Mallorca, and the red circles indicate the new strains isolated from Santa Pola. In blue we have indicated the old strains in the study in where triangles and circles represent the two different experimental sets of Mallorca and Santa Pola respectively. Both figures show that the old strains in both experiments do not behave homogeneously despite they are the same organisms.
(TIF)

Figure S5 **Distribution in the different metabolic pathways of the annotable metabolites responsible for the differences between old and new isolates in each of the analyzed datasets.** Metabolic classes corresponding to the different pathways are indicated.
(TIF)

Figure S6 **Diagrams showing the variability of the diversity Shannon index (A) and the coverage Good’s Index (B) in relation to the normalized data calculated from the Ward distances given in supplementary Figures S2 to S3.** Data was calculated for each dataset (pellet and SN2) of both experimental sets (Santa Pola and Mallorca). The blue bar indicates the 10% dissimilarity clustering threshold that gives the best compromise between diversity measures (between 1.5 to 2) and the coverage (around 90%). However these thresholds did not produce any model for which the clustering observed was statistically supported. The green bar indicates the 40% clustering threshold that in all cases produced a reliable statistical model supporting the clustering observed.
(TIF)

Figure S7 **Distribution in the different metabolic pathways of the annotable discriminative metabolites responsible for the clusters shown in Figure S5.** Metabolic classes corresponding to the different pathways are indicated.
(TIF)

Table S1 **Discriminative metabolites found in the different subsets of strains.**
(XLSX)
