# Supplementary figures and images for: High Metabolomic Microdiversity within Co-Occurring Isolates of the Extremely Halophilic Bacterium Salinibacter ruber
Source: PLoS One. 2013 May 31;8(5):e64701. doi: 10.1371/journal.pone.0064701 (PMC3669384; doi:10.1371/journal.pone.0064701)

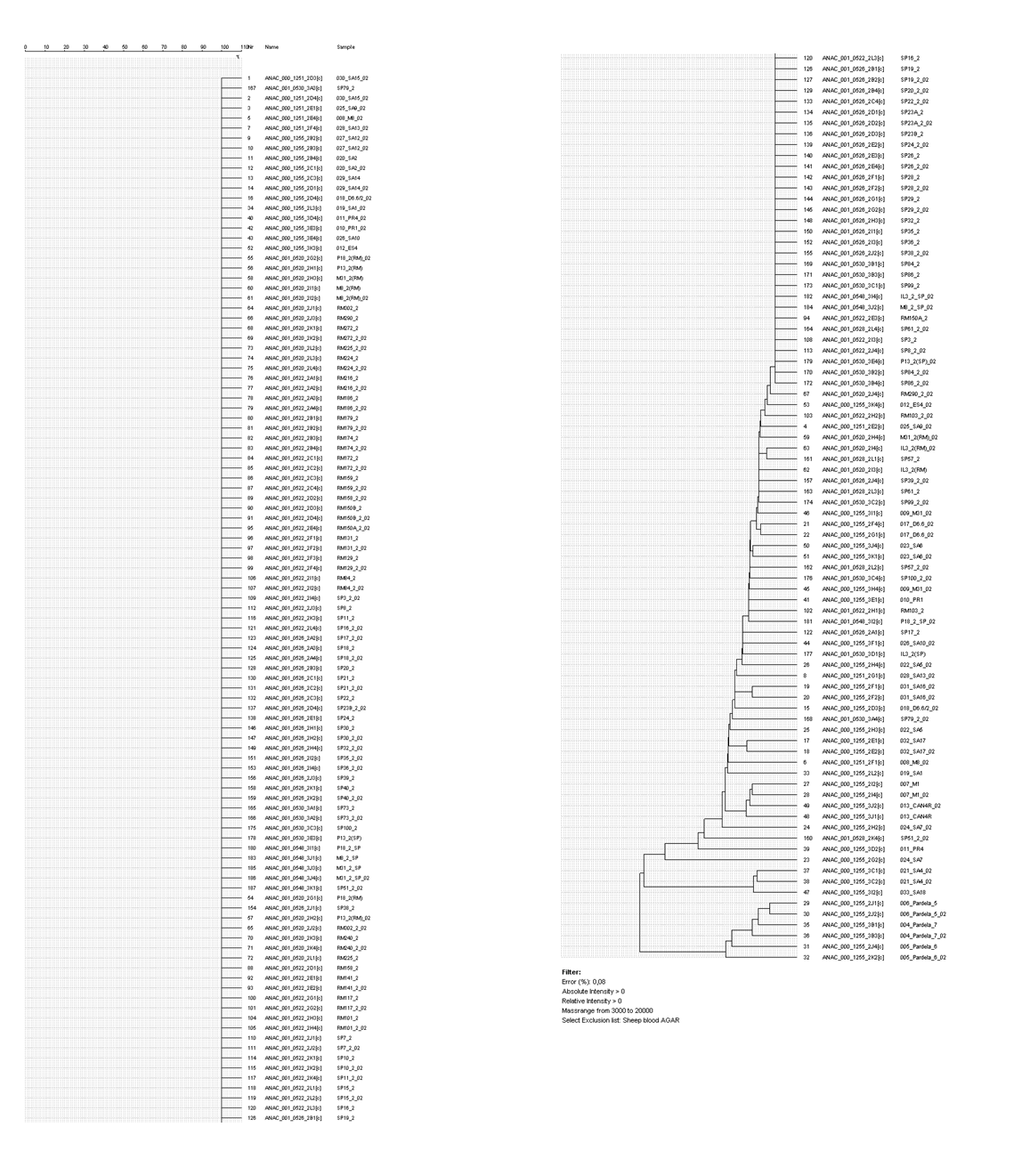

Supplement: Figure S1 — Dendrogram based on the Maldi-Tof profiles of all strains used in this work. Duplicates analyzed after one week of culture incubation are indicated by a _2 in the dendrogram. A group of 3 Halococcus sp. (Pardela 6 to 8) have been used as out-group for the analyses, as well as some additional unidentified isolates from S’Avall salterns (Mallorca) had been used as internal controls. S’Avall saltern isolates are indicated with the prefix SA (TIF) [file pone.0064701.s001.tif]

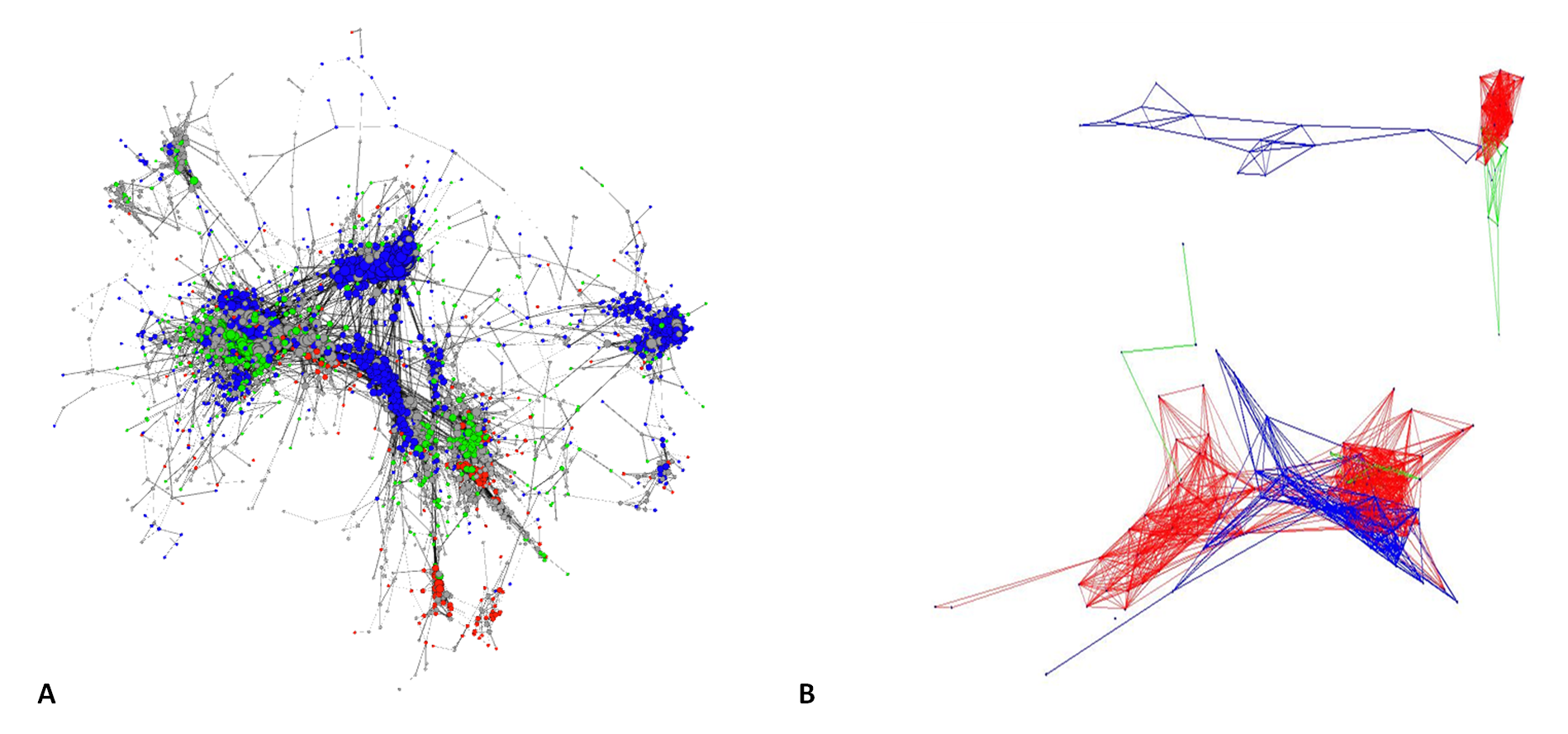

Supplement: Figure S2 — A: Mass Difference Network created from all annotated data Node Colors: Blue: Extracellular, Green: Intracellular, Red:Pellet. Colors were given only if the frequency of an m/z peak was 10 fold higher than the average mass frequency within the other classes. B: Correlation network created at threshold 0.90. Each nodes represent a sample; the closest the highest the correlation Regions are labeled as follows: extracellular blue, intracellular green, and pellet red. This approach individualized class specific subnetworks confirming thus the PCA grouping of the sample presented in Figure 2 . (TIF) [file pone.0064701.s002.tif]

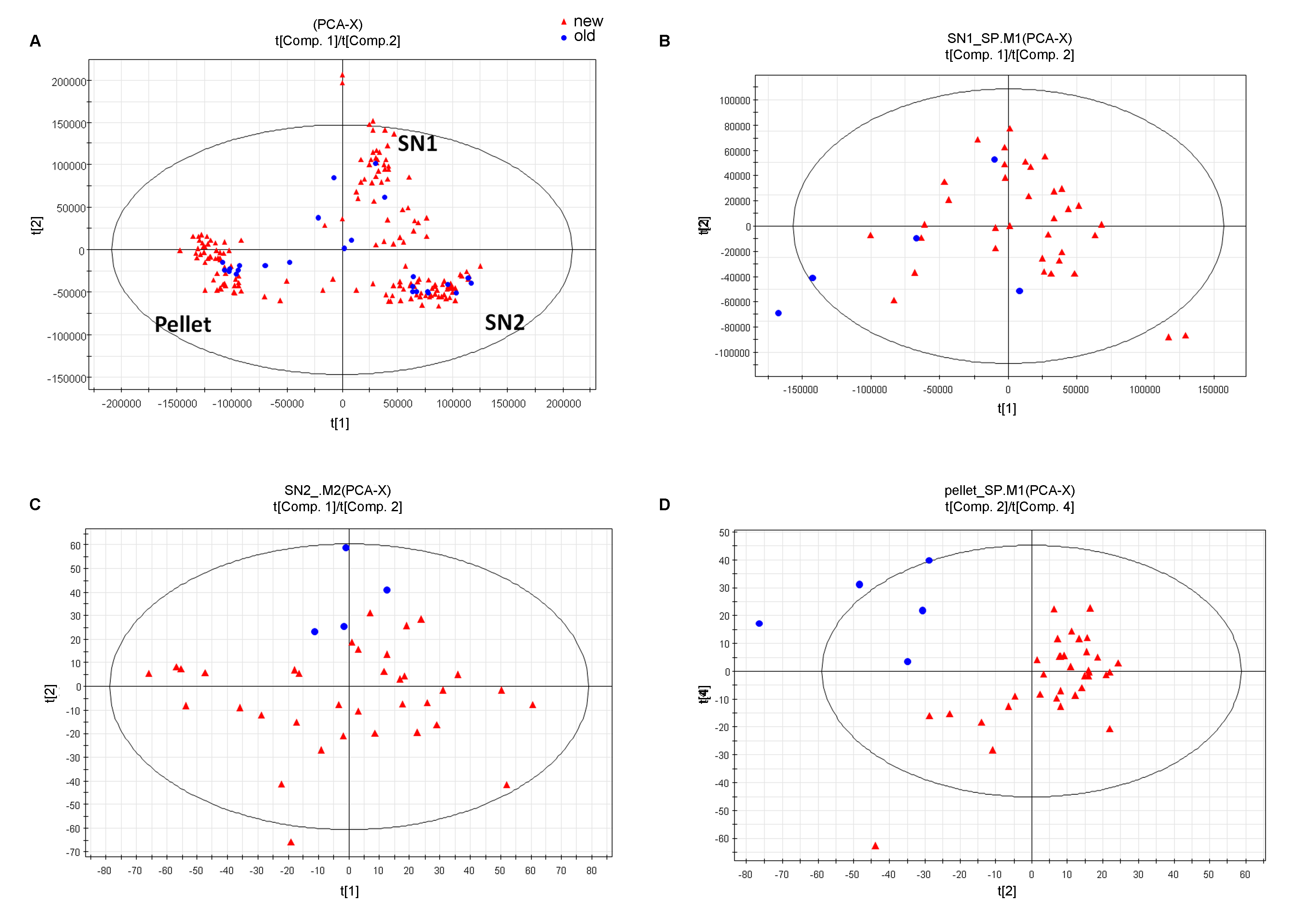

Supplement: Figure S3 — All the data together, in blue the different control samples and in red the new strains (A) As an example, the analyses for the Santa Pola isolates are shown: SN1 (B) SN2 (C) and pellet (C). Score scatter plots of all the isolates (A) from these we spit the data in 3 datasets to visualize that the dispersion of the control samples in SN1 (B) is greater than in the other datasets(C, D). 2012. (TIF) [file pone.0064701.s003.tif]

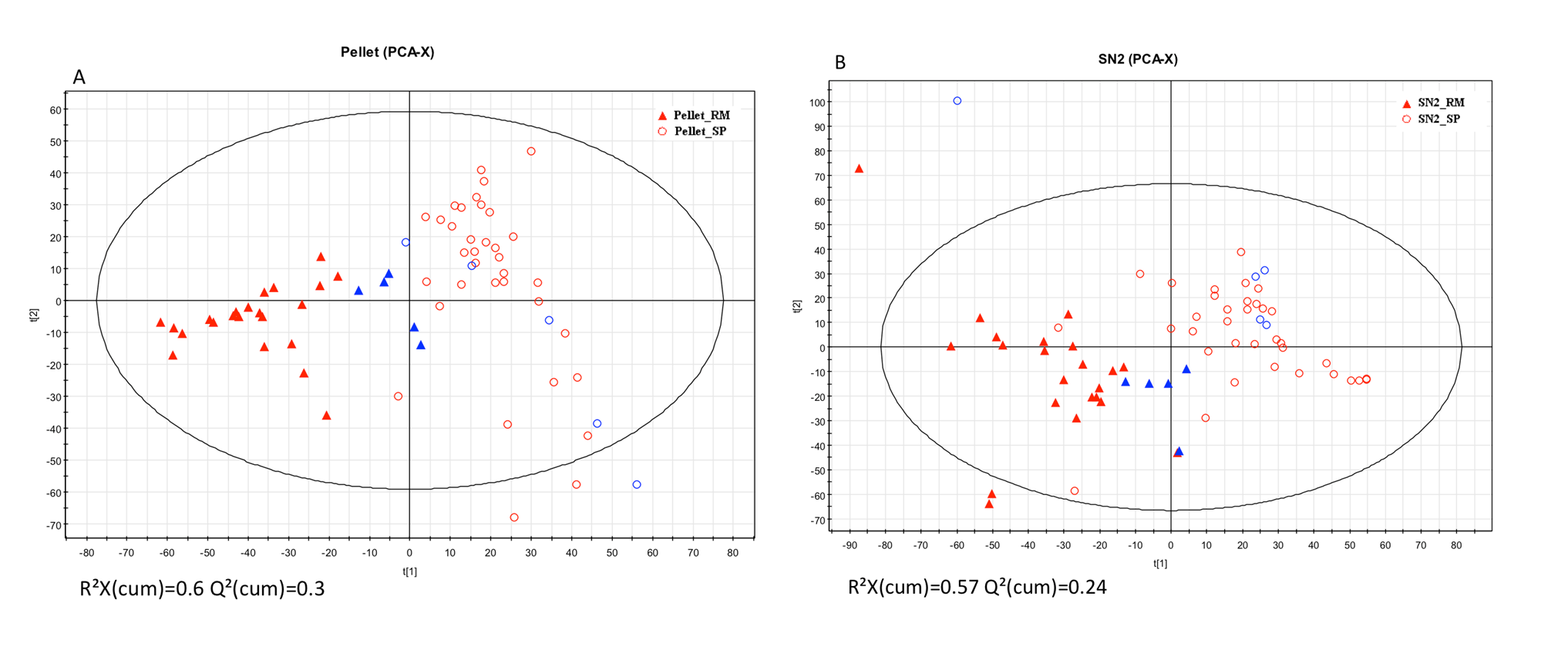

Supplement: Figure S4 — Unsupervised PCA analysis of each fraction (SN2 (A) and Pellet (B)) taking into account all samples obtained. Red triangles indicate the new strains of Mallorca, and the red circles indicate the new strains isolated from Santa Pola. In blue we have indicated the old strains in the study in where triangles and circles represent the two different experimental sets of Mallorca and Santa Pola respectively. Both figures show that the old strains in both experiments do not behave homogeneously despite they are the same organisms. (TIF) [file pone.0064701.s004.tif]

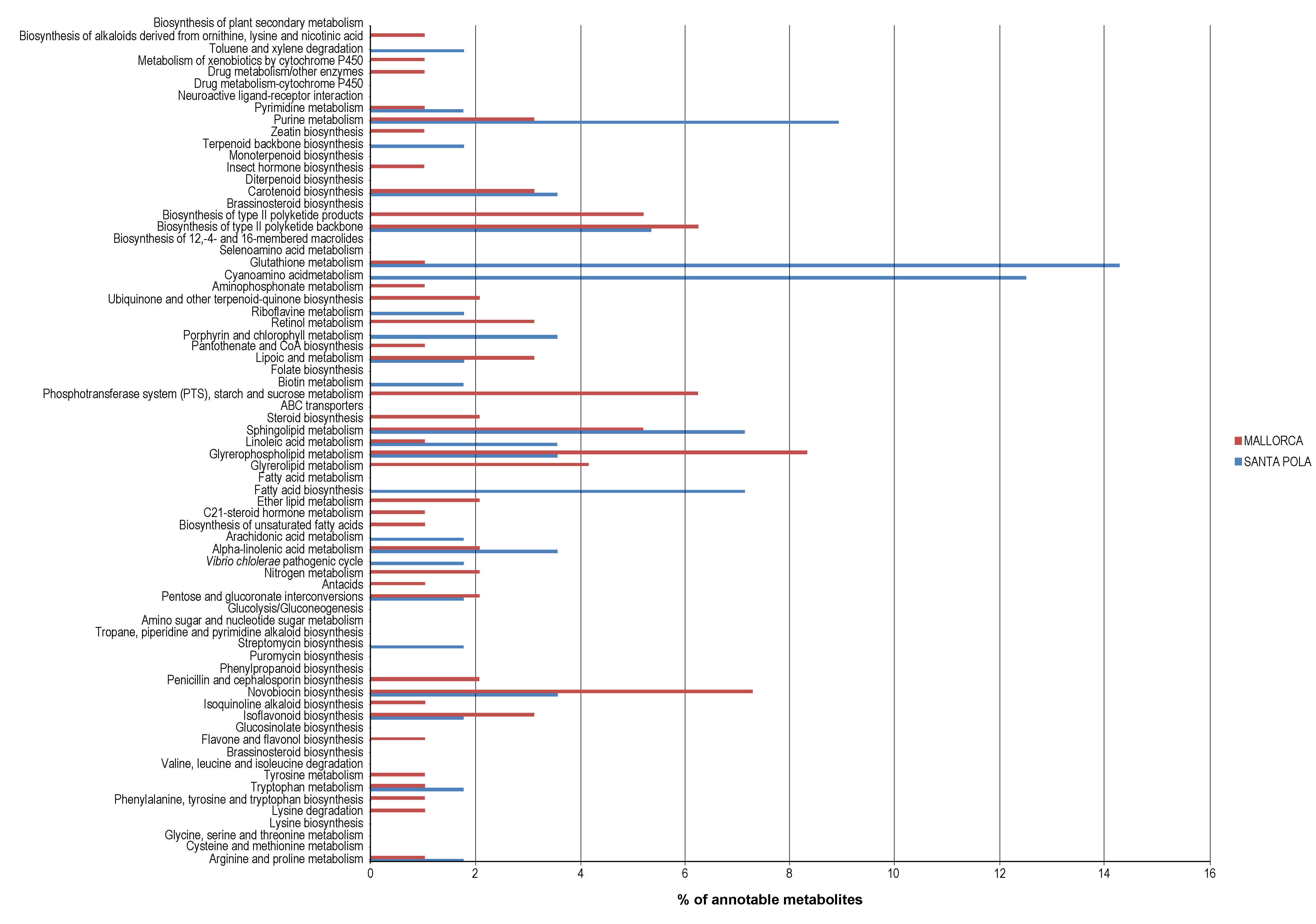

Supplement: Figure S5 — Distribution in the different metabolic pathways of the annotable metabolites responsible for the differences between old and new isolates in each of the analyzed datasets. Metabolic classes corresponding to the different pathways are indicated. (TIF) [file pone.0064701.s005.tif]

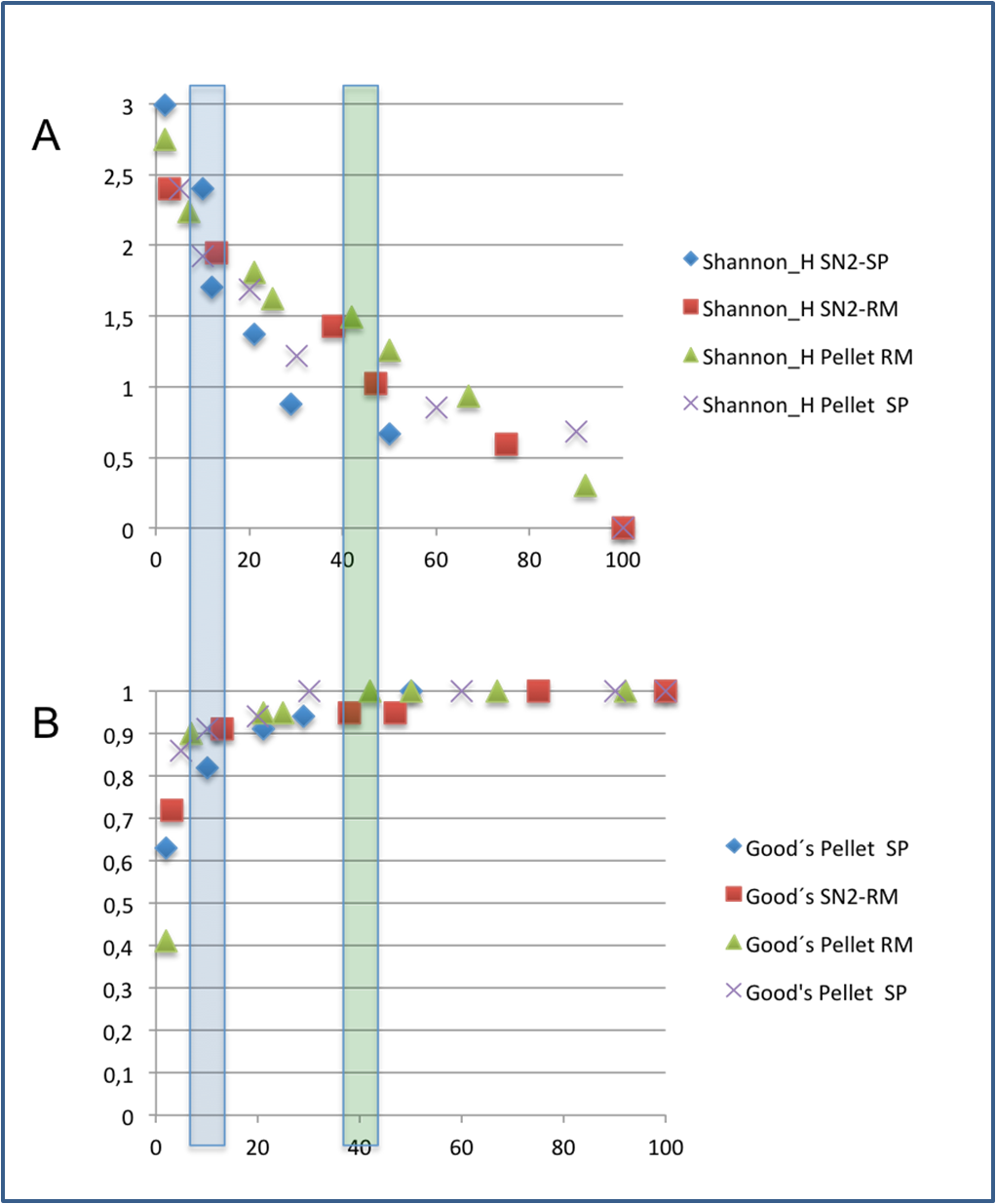

Supplement: Figure S6 — Diagrams showing the variability of the diversity Shannon index (A) and the coverage Good’s Index (B) in relation to the normalized data calculated from the Ward distances given in supplementary Figures S2 to S3. Data was calculated for each dataset (pellet and SN2) of both experimental sets (Santa Pola and Mallorca). The blue bar indicates the 10% dissimilarity clustering threshold that gives the best compromise between diversity measures (between 1.5 to 2) and the coverage (around 90%). However these thresholds did not produce any model for which the clustering observed was statistically supported. The green bar indicates the 40% clustering threshold that in all cases produced a reliable statistical model supporting the clustering observed. (TIF) [file pone.0064701.s006.tif]

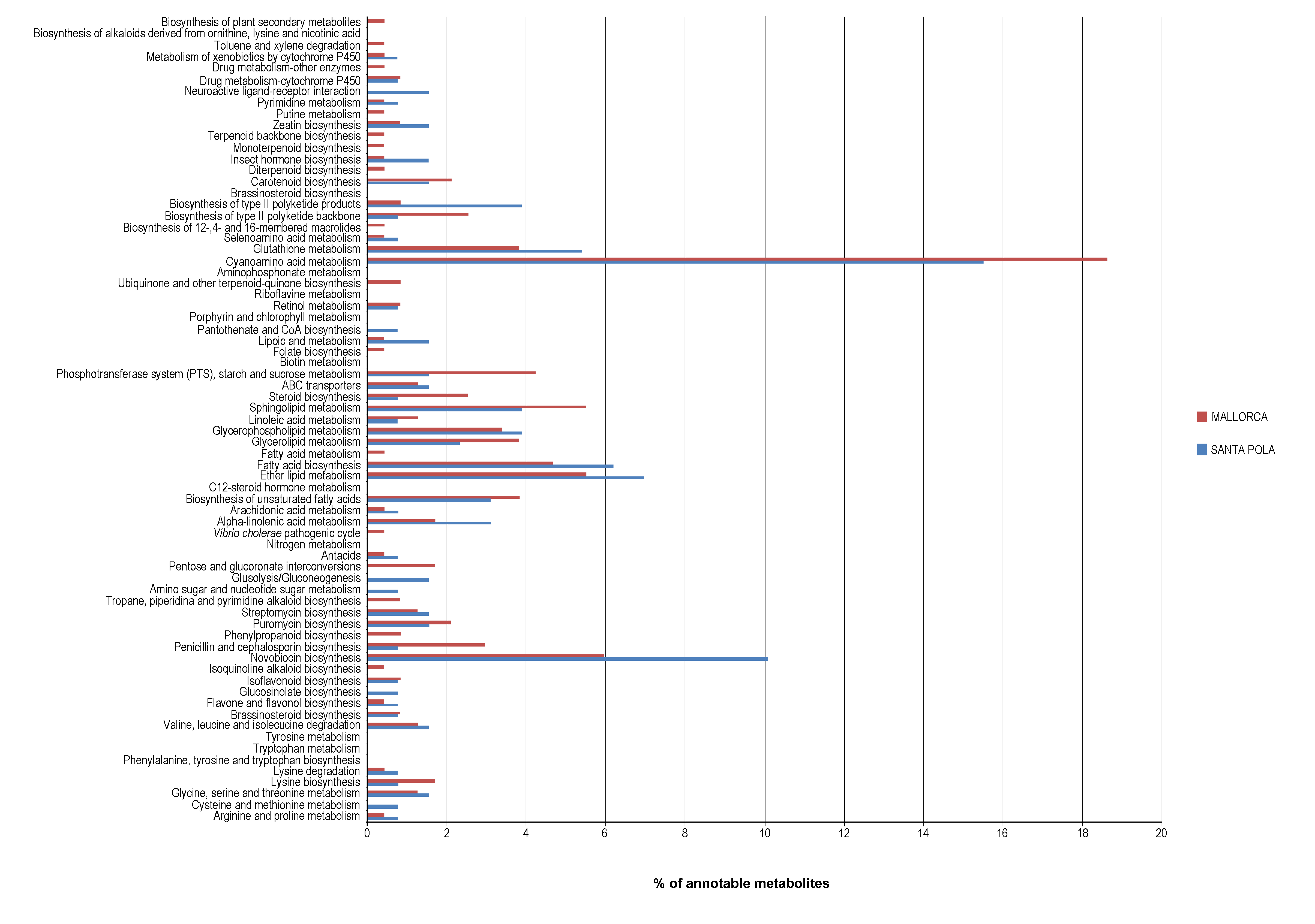

Supplement: Figure S7 — Distribution in the different metabolic pathways of the annotable discriminative metabolites responsible for the clusters shown in Figure S5. Metabolic classes corresponding to the different pathways are indicated. (TIF) [file pone.0064701.s007.tif]
